# Supplementary material for: Molecular mechanism of a potassium channel gating through activation gate-selectivity filter coupling
Source: Nat Commun. 2019 Nov 26;10:5366. doi: 10.1038/s41467-019-13227-w (PMC6879586; doi:10.1038/s41467-019-13227-w)
Supplement: Supplementary file 1 — Supplementary Information [file 41467_2019_13227_MOESM1_ESM.pdf]

SUPPLEMENTARY INFORMATION

Molecular mechanism of a potassium channel gating through activation gate-selectivity filter coupling

Authors: Kopec et al.

This PDF includes:

Supplementary Figures 1 to 22

Supplementary Tables 1 to 10

Supplementary References 1 to 3

Other Supplementary Information for this manuscript includes

Supplementary Movies 1 to 4

Supplementary Software

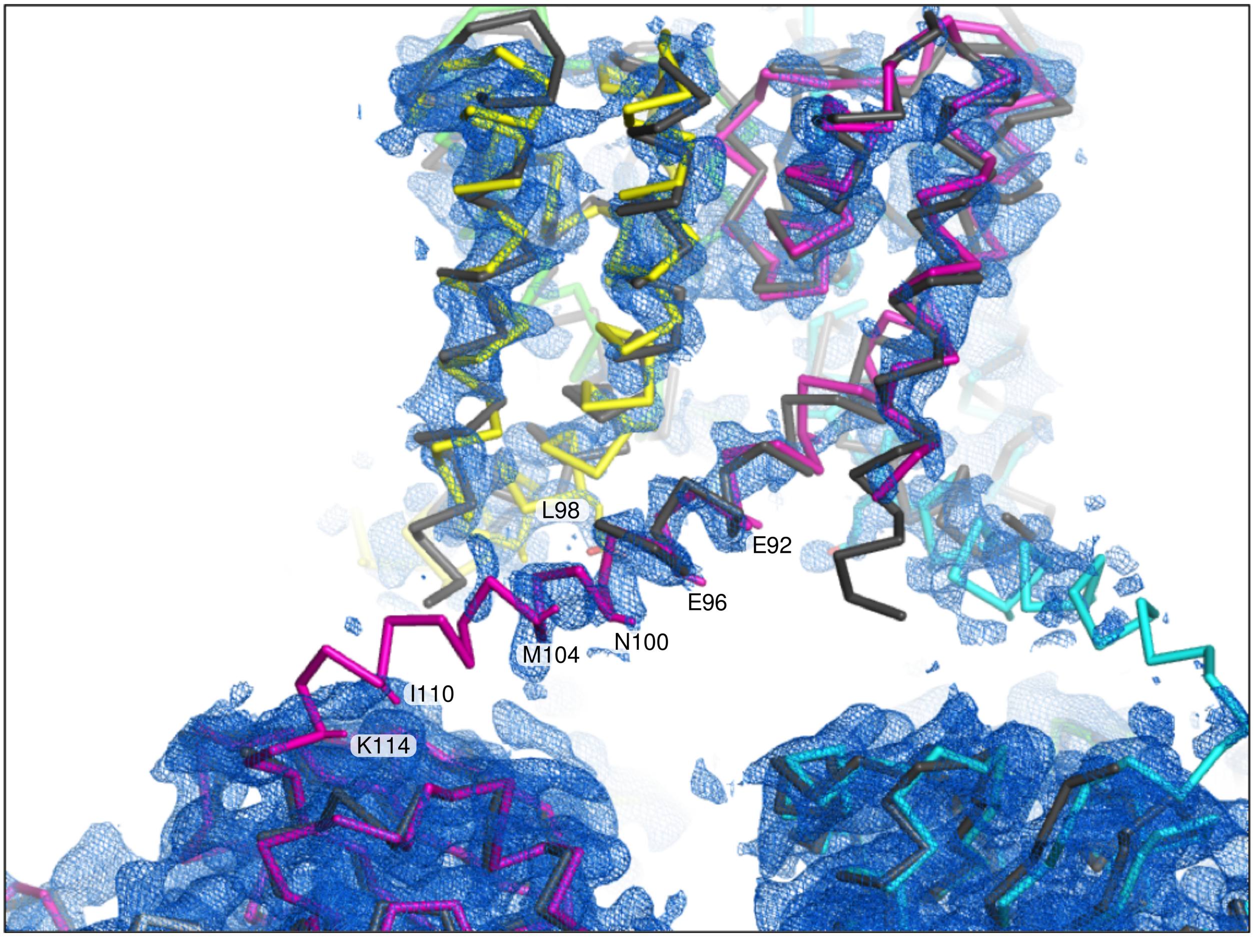

Supplementary Figure 1. Comparison of the pore regions of the completed full-length MthK structure (6OLY) with 1LNQ structure. 1LNQ structure is shown as gray C-alpha trace. 2Fo-Fc map (blue mesh, contoured at 0.8 σ) is superimposed on the structure, along with the 1LNQ structure (1), which is missing the segment corresponding to residues L99 through S115. The newly-resolved map defined the positions of main chain atoms corresponding to L99 through L106, and I110 through S115. Electron density corresponding to residues M107, G108, and L109 remains unresolved; however, these were placed using alpha-helical stereochemical restraints. The overall structure of the pore-linker-RCK module corresponded well with that observed in the structures of the related Slo1 Ca^{2+}-activated K (BK) channel and Slo2.2 Na-activated K channel (2, 3).

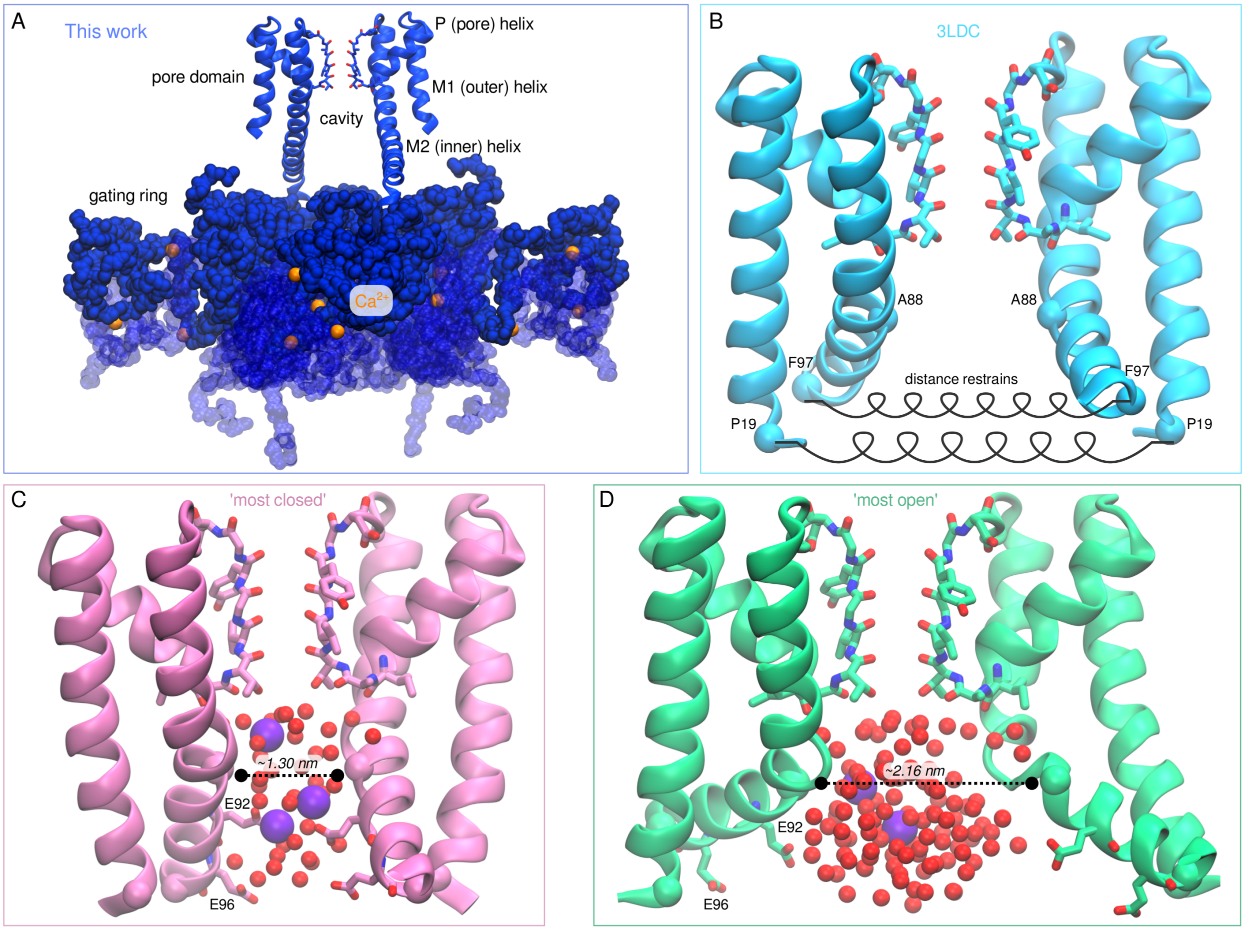

Supplementary Figure 2. Computational opening of MthK. (A) Crystal structure of a full length MthK channel. The transmembrane (TM) domain is linked to the cytoplasmic domains termed 'gating ring' (shown in blue surface) which bind Ca^{2+} ions (orange spheres). The conformational changes of the gating ring pull TM helices from the pore domain, which underlies gating transitions. **(B)** Scheme of the computational protocol used do generate more open and more closed states of MthK. Starting from the high resolution X-ray crystal structure (PDB ID: 3LDC), distance restraints were applied to CA atoms of residues F97 and P19, which resulted in opening and closing of TM helices. **(C)** and **(D)** The 'most closed' and 'most open' structures of MthK generated in this work, used in simulations called '4QE9-0.6' and '1LNQ+0.6' (see Table 2 in the main text). The distance between CA atoms of A88 is indicated. Negatively charged glutamates E92 and E96 as well as residues forming the SF are shown as sticks, K^+ ions as purple spheres and water molecules in the cavity as red spheres.

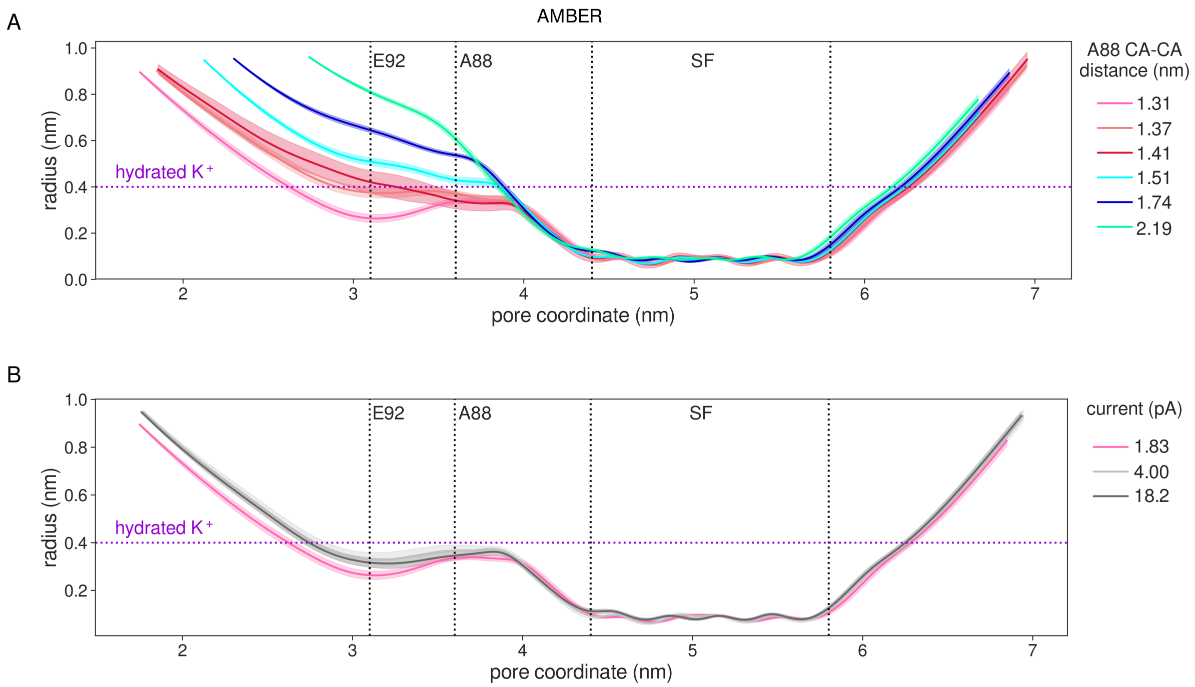

Supplementary Figure 3. Pore radius profiles calculated for simulations performed with the AMBER force field. (A) Profiles for several simulations with different levels of the AG opening. Colors correspond to the Fig 3 from the main text. **(B)** Comparison of profiles for simulations in which the distance restraints were applied to the helix ends (pink curve) or to the distance between CA atoms of T59 residues (see Fig 2 in the main text). Error bars represent 95% confidence intervals. Source data are available as a Source Data file.

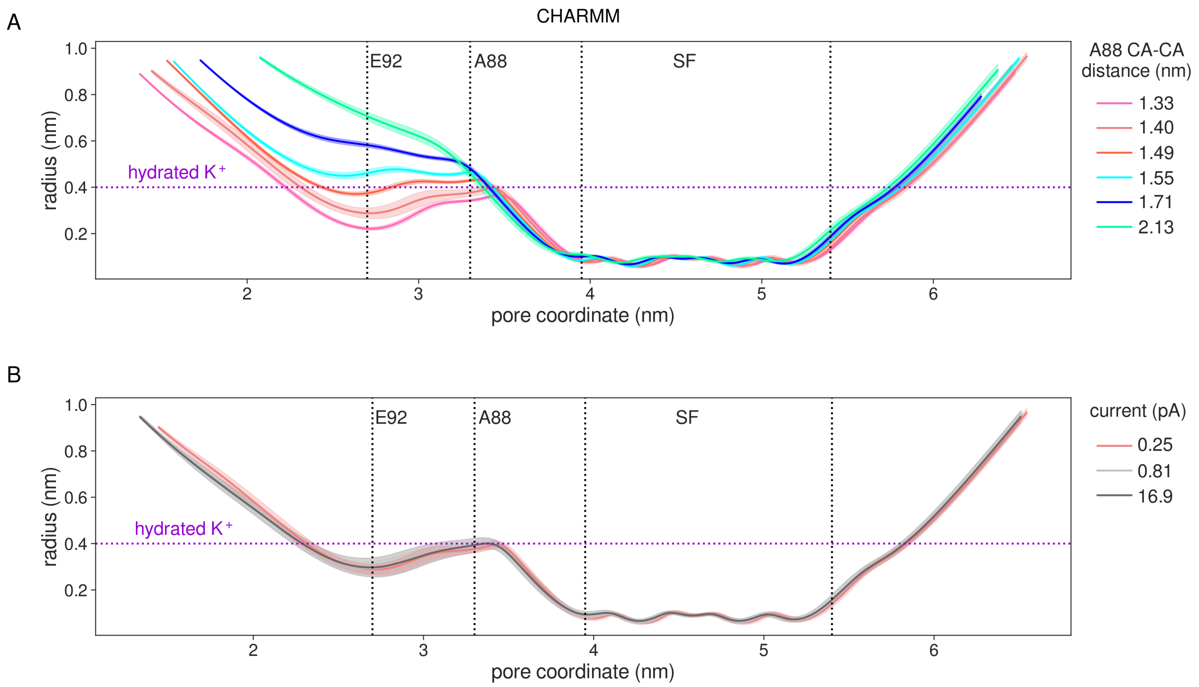

Supplementary Figure 4. Pore radius profiles calculated for simulations performed with the CHARMM force field. (A) Profiles for several simulations with different levels of the AG opening. Colors correspond to the Fig 3 from the main text. **(B)** Comparison of profiles for simulations in which the distance restraints were applied to the helix ends (pink curve) or to the distance between CA atoms of T59 residues (see Fig 2 in the main text). Error bars represent 95% confidence intervals. Source data are available as a Source Data file.

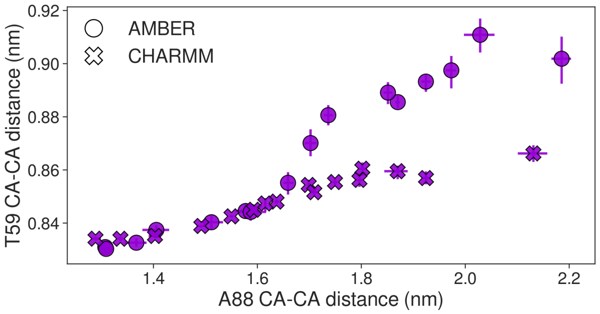

Supplementary Figure 5. Correlations between the AG opening (A88 CA-CA distance) and the SF gate opening (T59 CA-CA distance). Error bars represent 95% confidence intervals. Source data are available as a Source Data file.

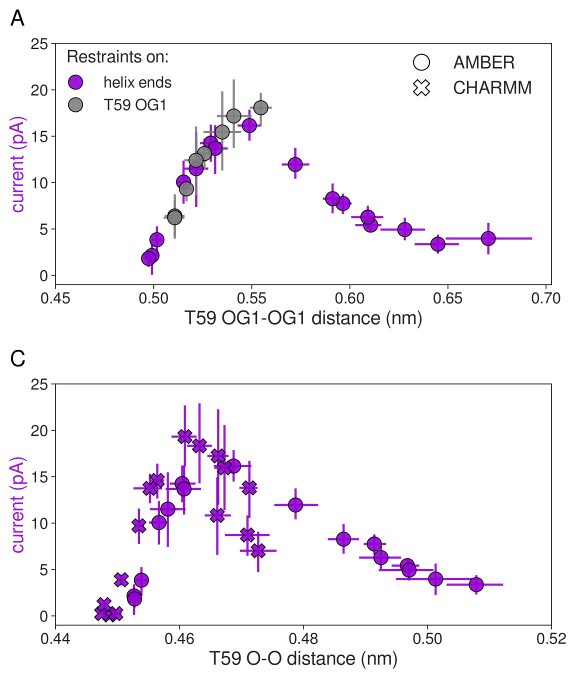

Supplementary Figure 6. Outward K$^+$ current as a function of T59 opening. (A) and **(B)** Outward K$^+$ current variations when the T59 OG-OG1 distance is used an opening coordinate, for AMBER and CHARMM force fields, respectively. Purple markers represent the same data as in Fig 2 B in the main text. Grey markers show the data obtained when the distance restraints were applied directly between the T59 OG1 atoms. **(C)** Outward K$^+$ current through MthK at 300 mV as a function of channel opening when the T59 O-O distance is used as an opening coordinate. Error bars represent 95% confidence intervals. Source data are available as a Source Data file.

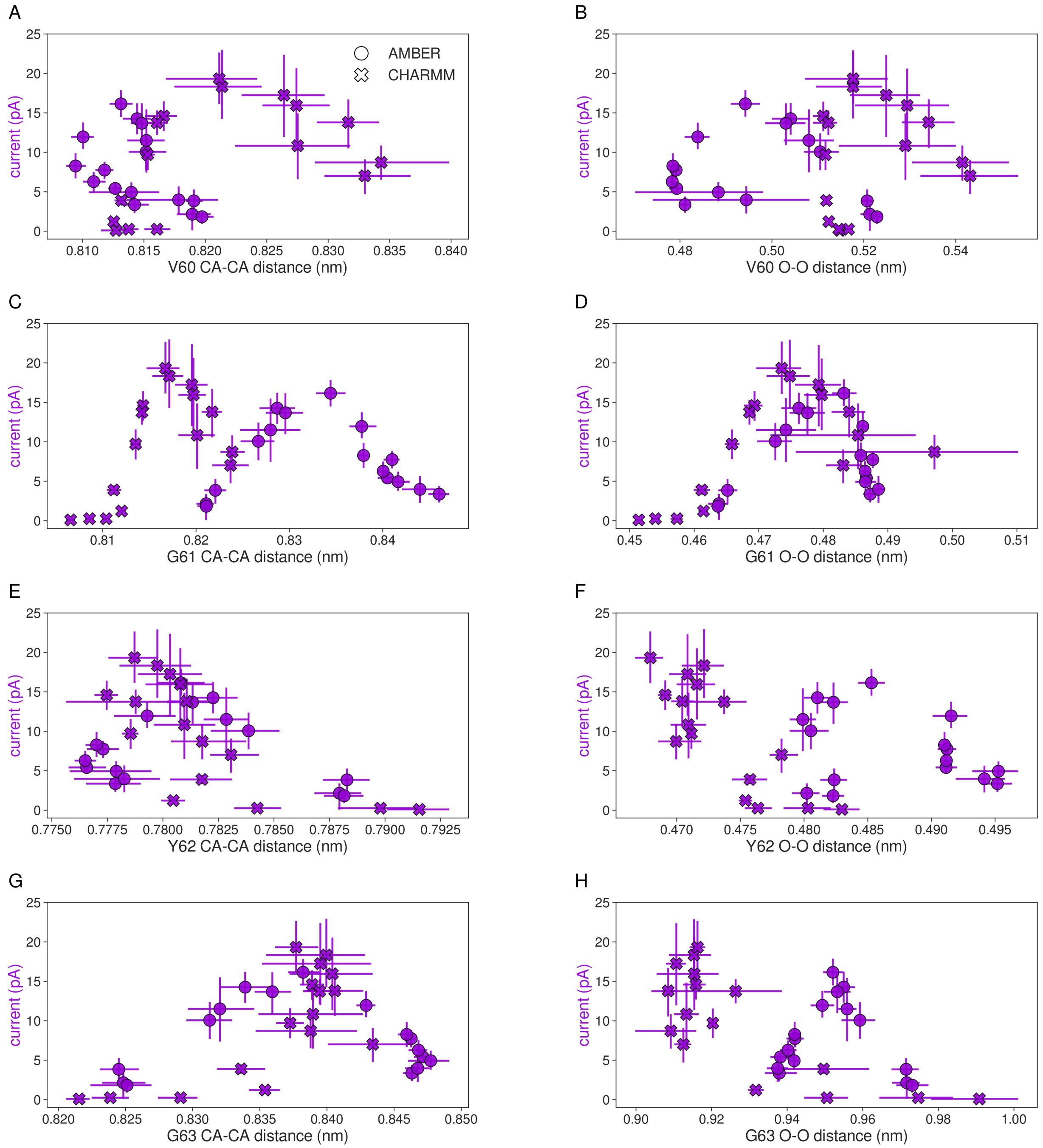

Supplementary Figure 7. Outward K^+ current as a function of the SF opening. Outward K^+ current variations when CA-CA or O-O distances of V60 ((**A)** and **(B)**), G61 ((**C)** and **(D)**), Y62 ((**E)** and **(F)**) and G63 ((**G)** and **(H)**) are used as opening coordinates, respectively. Error bars represent 95% confidence intervals. Source data are available as a Source Data file.

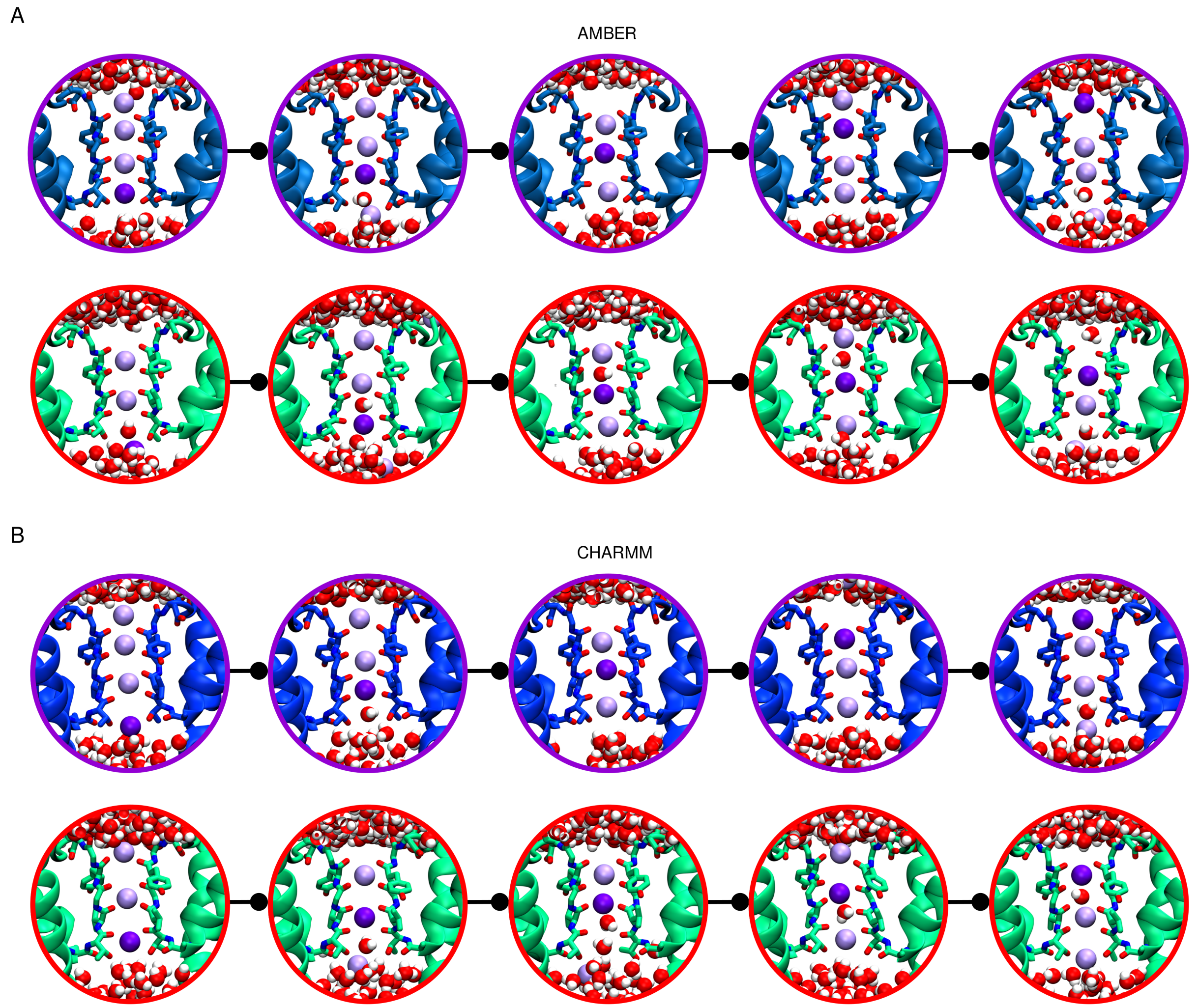

Supplementary Figure 8. Snapshots from MD simulations showing ion permeation at different opening levels. For both force fields (**(A)** – AMBER, **(B)** – CHARMM)) K^+ ions permeate MthK without accompanying water molecules (i.e. through the 'direct knock-on' mechanism) at opening levels for which the highest currents are observed (upper rows), whereas for more open channels water molecules start to permeate the channel as well (bottom rows). The SF is shown in sticks, K^+ ions as purple spheres (darker color marks the permeating ion) and water molecules as red and white spheres. Channel coloring corresponds to colors used in Fig 3 in the main text, marking different levels of opening.

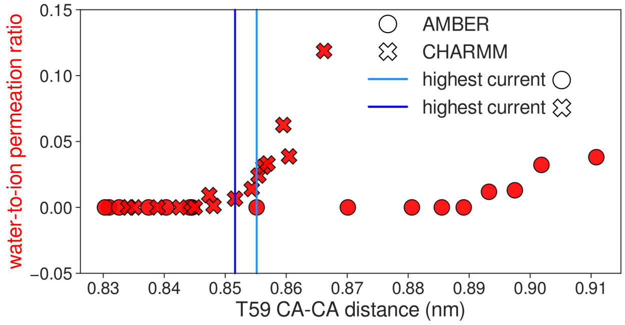

Supplementary Figure 9. Number of water molecules permeating MthK per ion (water-to-ion permeation ratio) as a function of channel opening. For both force fields, water molecules do not permeate the channel for small and moderate opening (water-to-ion ratio almost 0), whereas for more open channels water molecules can occasionally be transported alongside K^+ ions. Source data are available as a Source Data file.

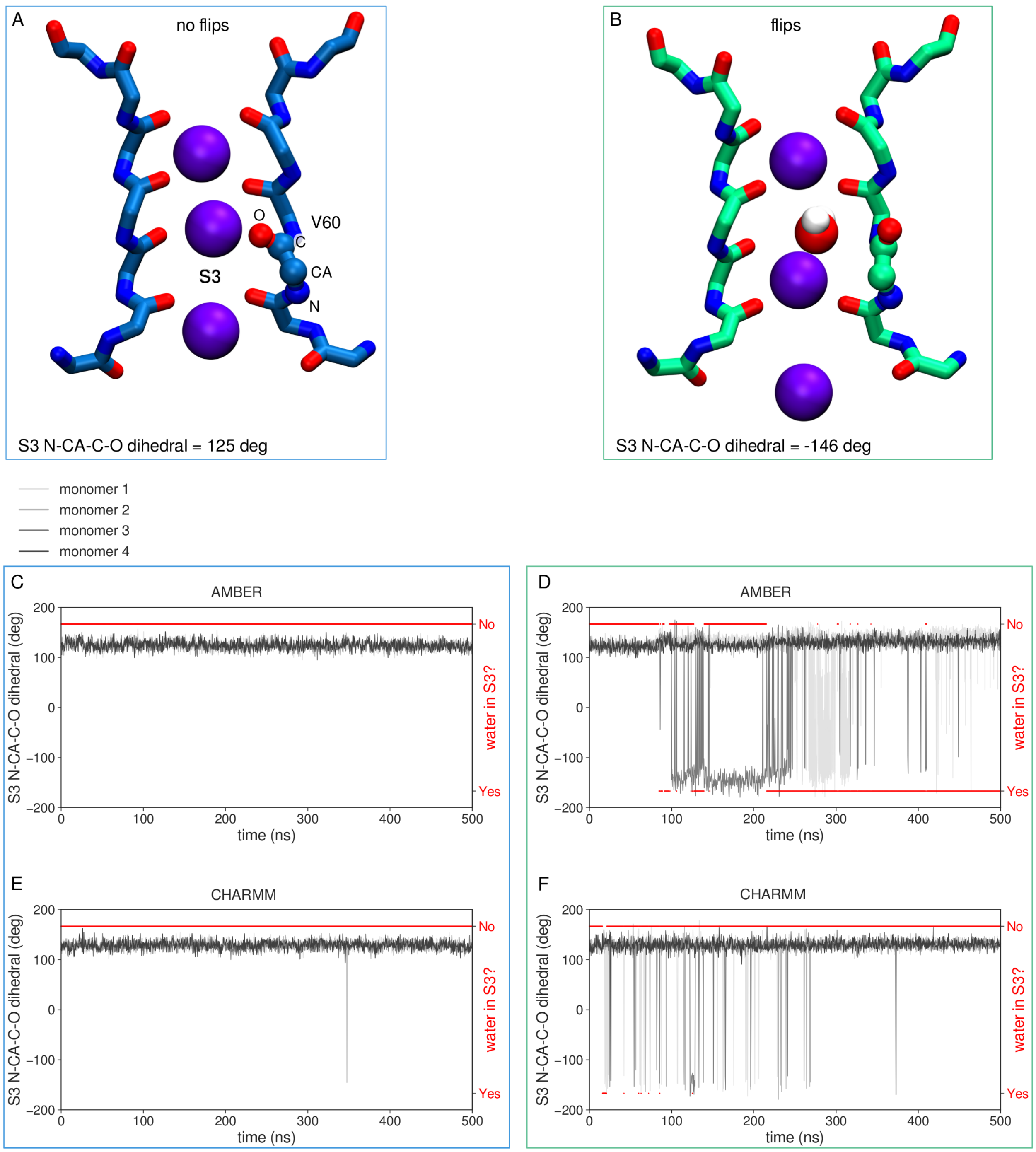

Supplementary Figure 10. The correlation between V60 carbonyl flipping and the water presence in the S3 ion binding site. Non-flipped **(A)** and flipped **(B)** conformations of V60, together with the N-CA-C-O dihedral used to distinct between these conformations. Backbone of the SF forming residues are shown in sticks, K^+ ions as purple spheres and water molecules as red and white spheres. Channel coloring corresponds to Figure 3 in the main text. Examples of trajectory traces in which water molecules

either do not enter (**(C)** and **(E)**) or do enter (**(D)** and **(F)**) the SF, corresponding to different levels of channel opening. In the latter case, V60 flipping correlates with the water presence in S3 ion binding site.

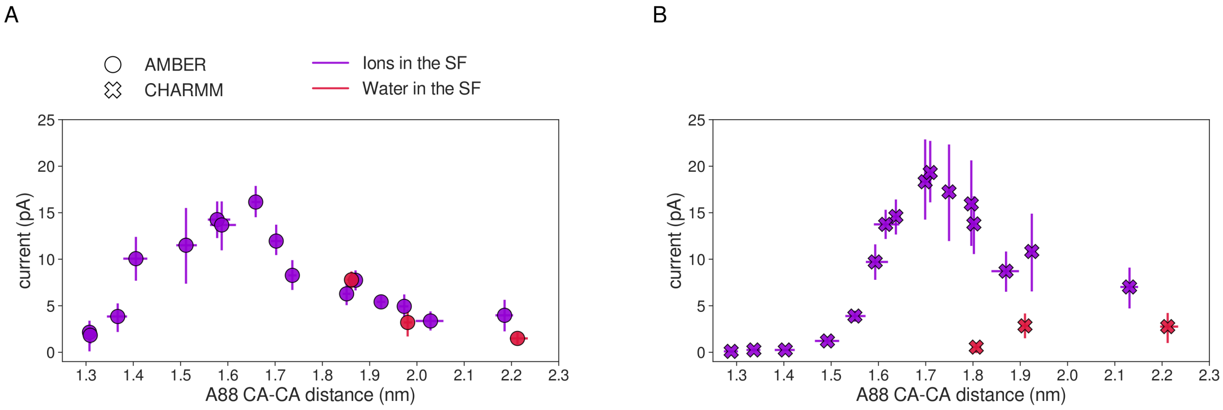

Supplementary Figure 11. Outward K$^+$ current as a function of A88 opening with different starting occupancies of the SF. (A) and **(B)** Outward K$^+$ current variations when A88 CA-CA distance is used an opening coordinate, for AMBER and CHARMM force fields, respectively. Purple marks represent the same data as in the Figure 2 B in the main text. Red marks represent data obtained from simulations started with water molecules present in the SF. Error bars represent 95% confidence intervals. Source data are available as a Source Data file.

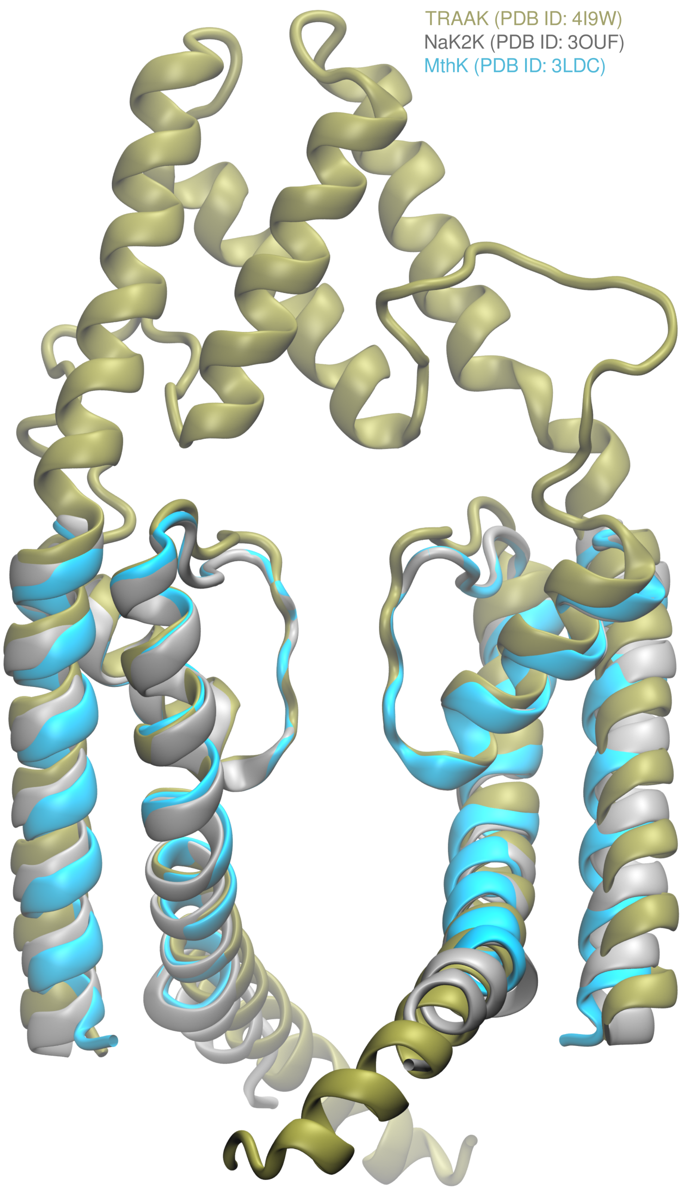

Supplementary Figure 12. Overlay of crystal structures of MthK, TRAAK and NaK2K channels.

All three channels share a common architecture of transmembrane helices and the selectivity filter.

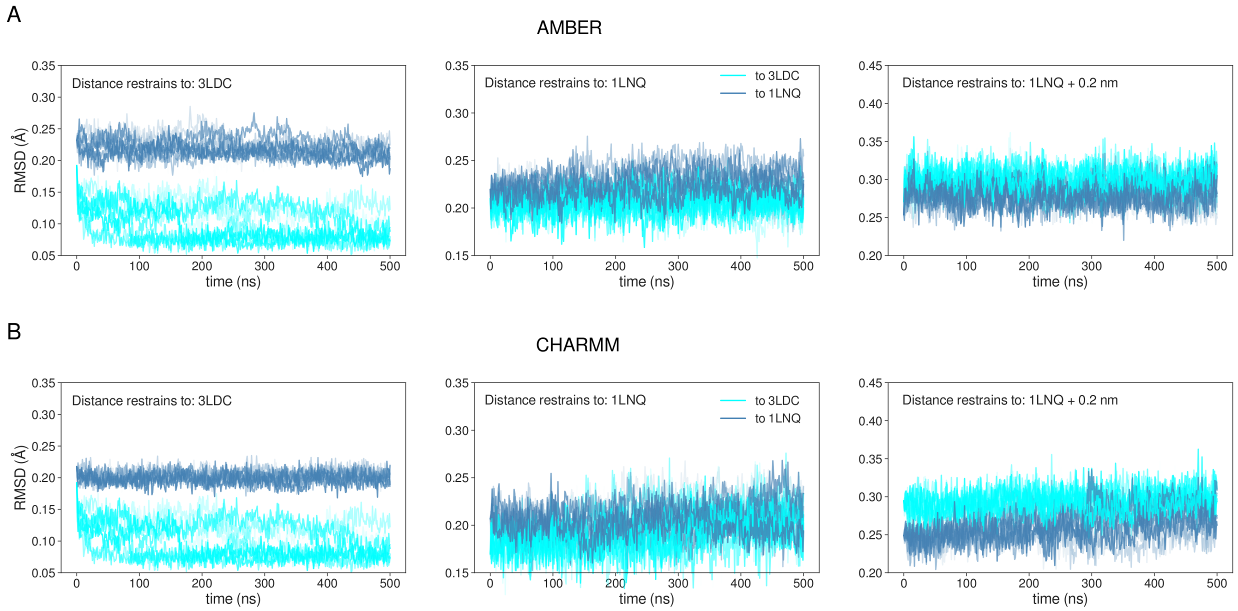

Supplementary Figure 13. Root mean square displacement (RMSD) of M2 helix ends. (A) and **(B)** RMSD profiles for all simulations from a given set of simulations (3LDC, 1LNQ, 1LNQ + 0.2, see Table 2 in the main text), for AMBER and CHARMM force fields, respectively. Colors represent which structure was used as a reference (3LDC or 1LNQ) for RMSD calculations. Source data are available as a Source Data file.

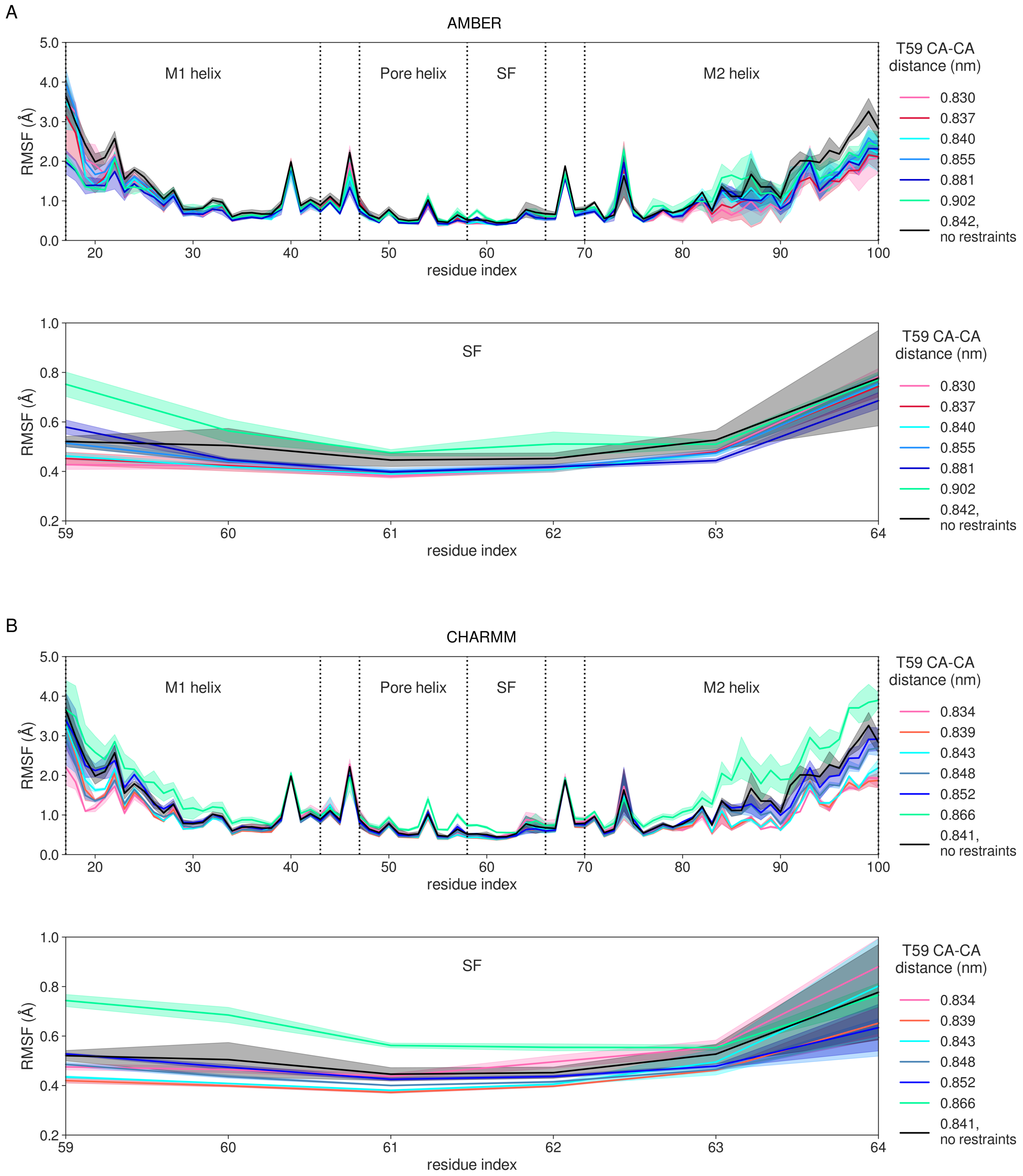

Supplementary Figure 14. Root mean square fluctuations (RMSF) per residue in MthK simulations. (A) and **(B)** RMSF profiles for AMBER and CHARMM force fields, respectively. Colors correspond to Figure 3 in the main text. Black lines correspond to simulations started from the 3LDC structure, but without any distance restraints applied. Importantly, the outward currents in simulations with distance restraints to the 3LDC structure (i.e. cyan lines) and without any restraints (i.e. black lines) are very similar: 11.5 ± 2.5 pA and 14.5 ± 1.5 pA in AMBER and 3.6 ± 0.5 pA and 4.9 6 ± 1.4 pA in

CHARMM, respectively. Error bars represent 95% confidence intervals. Source data are available as a Source Data file.

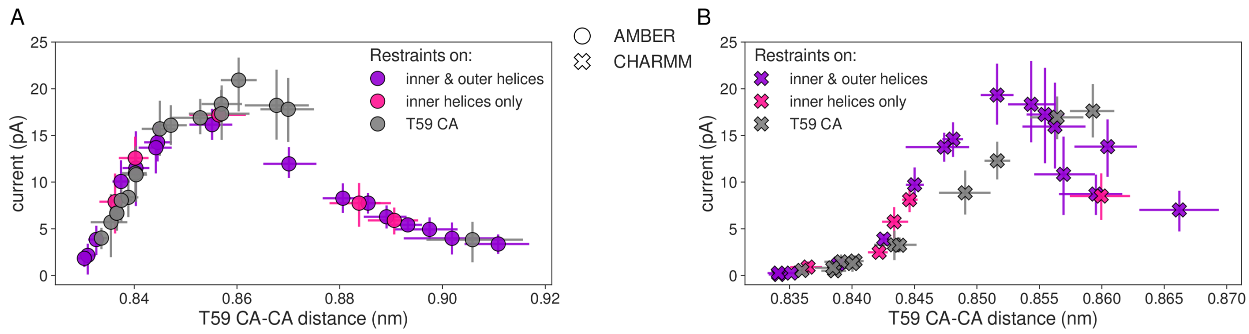

Supplementary Figure 15. Outward K^+ current as a function of T59 opening. (A) and **(B)** Outward K^+ current variations when T59 CA-CA distance is used an opening coordinate, for AMBER and CHARMM force fields, respectively. Purple and grey markers represent the same data as in Figure 2 B and C in the main text. Magenta markers show the data obtained when the distance restraints were applied to the ends of inner (M2) helices only. Error bars represent 95% confidence intervals. Source data are available as a Source Data file.

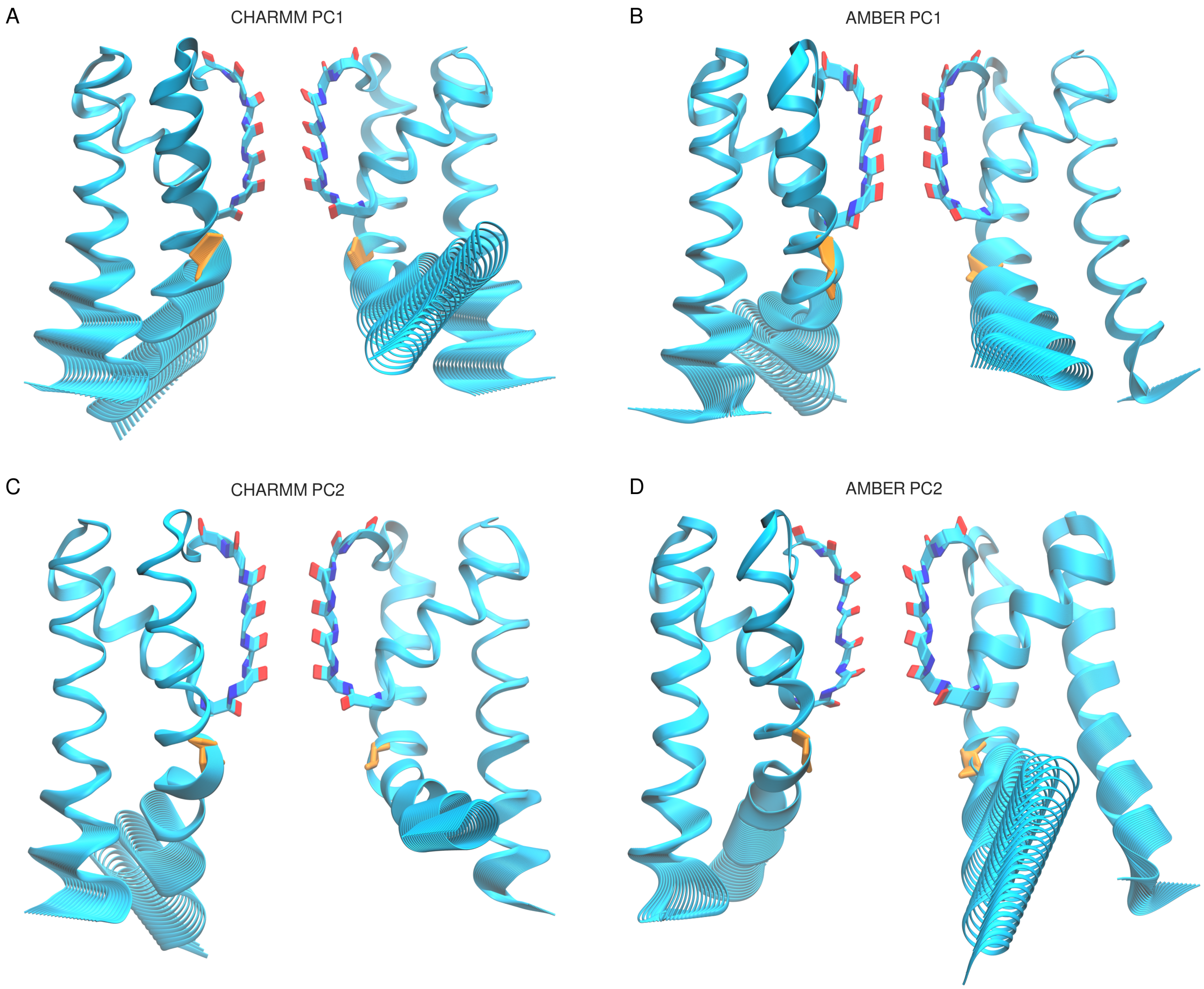

Supplementary Figure 16. Visualization of the first two PC, for the AMBER and CHARMM force fields.

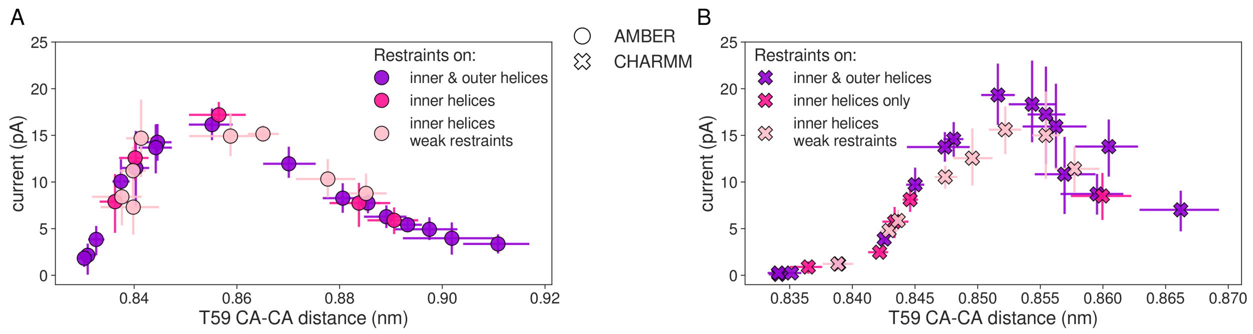

Supplementary Figure 17. Outward K^+ current as a function of T59 opening. (A) and **(B)** Outward K^+ current variations when T59 CA-CA distance is used an opening coordinate, for AMBER and CHARMM force fields, respectively. Purple markers represent the same data as in Figure 2 B in the main text. Magenta markers show the data obtained when the distance restraints were applied to the ends of inner (M2) helices only. Light pink markers show the data obtained when the distance restraints were applied to the ends of inner (M2) helices only and with the reduced force constant. Error bars represent 95% confidence intervals. Source data are available as a Source Data file.

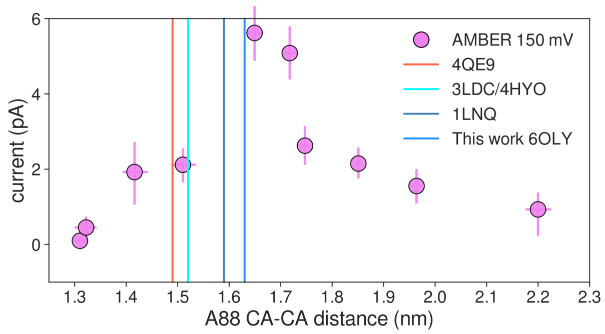

Supplementary Figure 18. Outward K^+ current as a function of A88 opening. Outward K^+ current variations at reduced (150 mV) voltage when A88 CA-CA distance is used an opening coordinate, for the AMBER force field. Error bars represent 95% confidence intervals. Source data are available as a Source Data file.

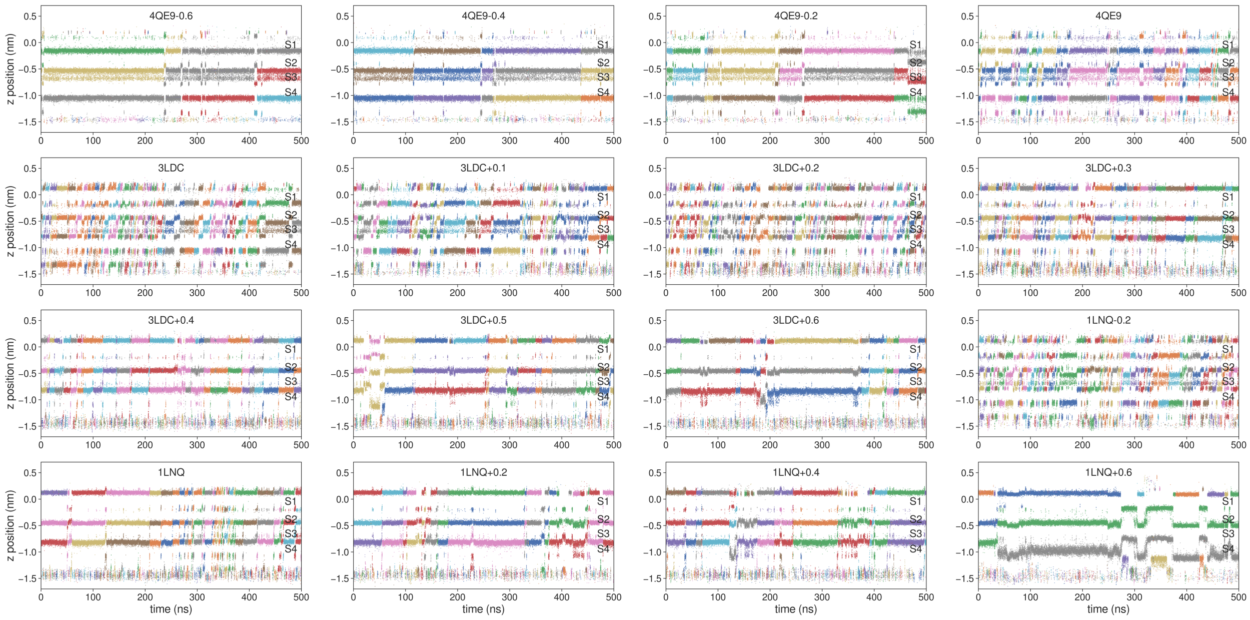

Supplementary Figure 19. Example traces of potassium ions inside the SF of MthK in simulations with the AMBER force field.

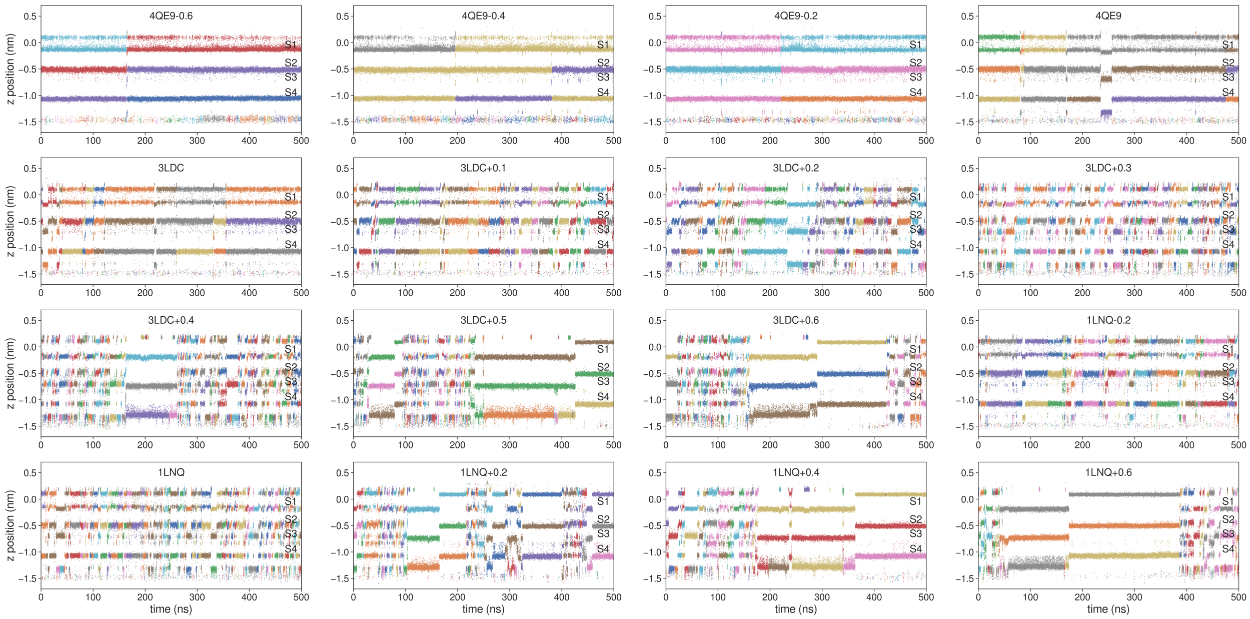

Supplementary Figure 20. Example traces of potassium ions inside the SF of MthK in simulations with the CHARMM force field.

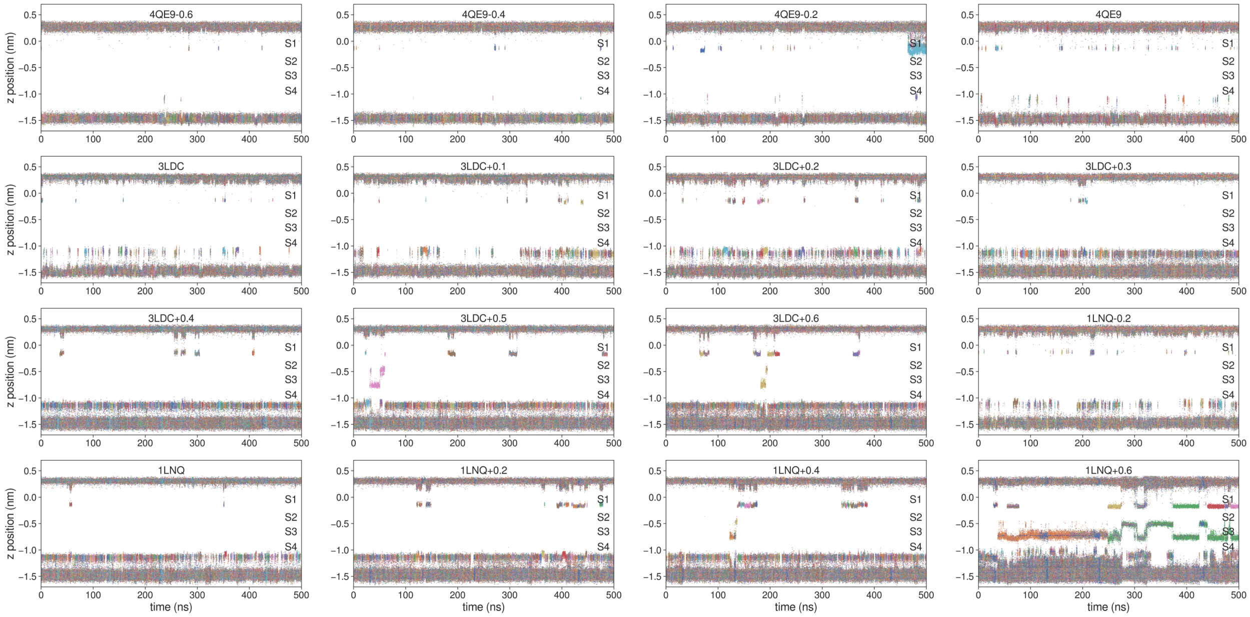

Supplementary Figure 21. Example traces of water molecules inside the SF of MthK in simulations with the AMBER force field.

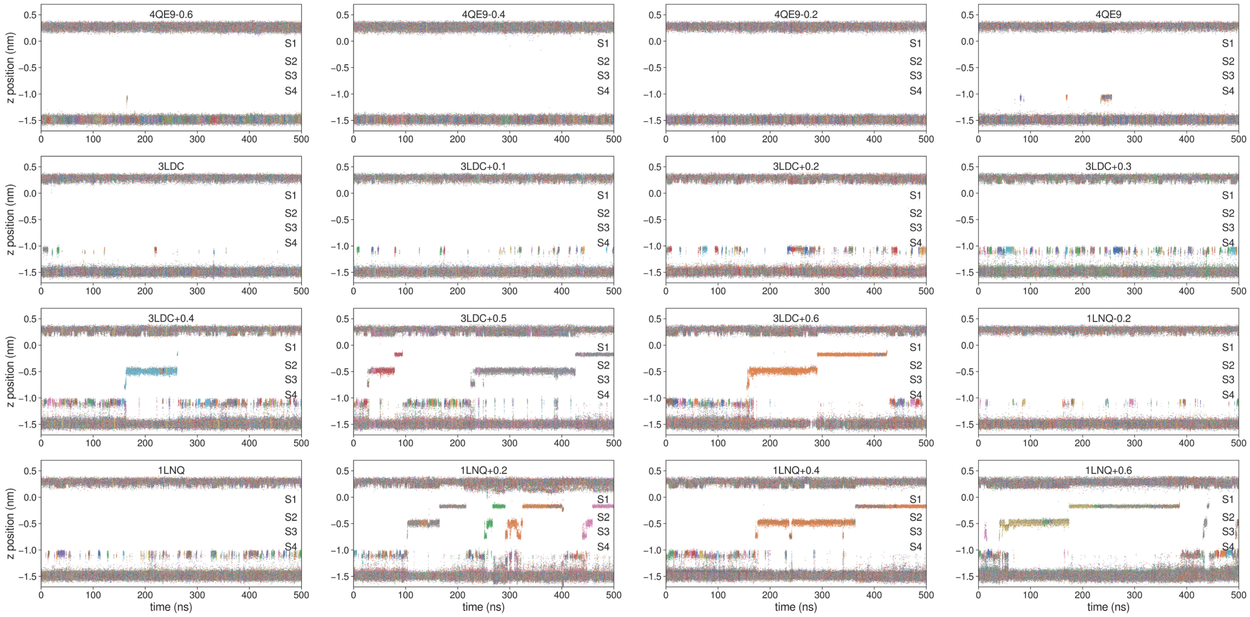

Supplementary Figure 22. Example traces of water molecules inside the SF of MthK in simulations with the CHARMM force field.

Supplementary Table 1. List of simulated systems (T59 CA restrains).

System name	Restrained T59 CA-CA distance (opposite subunits) (nm)	Distance restraint force constant (kJ/mol/nm2)
4QE9-0.6	0.833	500
4QE9-0.2	0.835	500
3LDC	0.843	500
1LNQ	0.852	500
0.78	0.78	500
0.80	0.800	500
0.825	0.825	500
0.865	0.865	500
0.885	0.885	500
0.910	0.910	500
0.930	0.930	500
0.950	0.950	500
0.970	0.970	500
0.990	0.990	500
0.995	0.995	500
1.00	1.00	500
1.01	1.01	500
1.05	1.05	500

Supplementary Table 2. List of simulated systems (T59 OG1 restrains).

System name	Restrained T59 OG1-OG1 distance (opposite subunits) (nm)	Distance restraint force constant (kJ/mol/nm2)
4QE9-0.2	0.833	500
3LDC	0.835	500
3LDC+0.2	0.843	500
3LDC+0.4	0.852	500
0.46	0.460	500
3LDC+0.5	0.800	500
0.48	0.480	500
0.57	0.570	500
0.61	0.610	500
0.63	0.630	500
0.65	0.650	500

Supplementary Table 3. List of simulated systems (I84Amutant).

System name	Restrained P19 CA-CA distance (opposite subunits) (nm)	Restrained P19 CA-CA distance (adjacent) (nm)	Restrained F97 CA-CA distance (opposite subunits) (nm)	Restrained F97 CA-CA distance (adjacent subunits) (nm)	Distance restraint force constant (kJ/mol/nm2)
4QE9-0.6	2.87	1.85	2.78	1.79	1000
4QE9-0.4	3.07	2.05	2.98	1.99	1000
4QE9-0.2	3.27	2.25	3.18	2.19	1000
3LDC-0.1	3.5	2.15	3.08	2.09	1000
3LDC	3.6	2.55	3.66	2.6	1000
3LDC+0.1	3.7	2.65	3.76	2.7	1000
3LDC+0.2	3.8	2.75	3.86	2.8	1000
3LDC+0.3	3.9	2.85	3.96	2.9	1000
3LDC+0.5	4.1	3.05	4.16	3.1	1000
1LNQ	4.0	2.8	4.3	3.0	1000

Supplementary Table 4. List of simulated systems (PLS-FMA).

System name	Restrained F97 CA-CA distance (opposite subunits) (nm)	Restrained F97 CA-CA distance (adjacent subunits) (nm)	Distance restraint force constant (kJ/mol/nm2)
4QE9-0.4	2.98	1.99	1000
3LDC	3.66	2.6	1000
3LDC+0.2	3.86	2.8	1000
1LNQ	4.16	3.1	1000
1LNQ+0.6	4.60	3.40	1000

Supplementary Table 5. List of simulated systems (MthK without restraints).

System name	Distance restraint force constant (kJ/mol/nm2)
3LDC	0

Supplementary Table 6. List of simulated systems (weaker distance restraints).

System name	Restrained F97 CA-CA distance (opposite subunits) (nm)	Restrained F97 CA-CA distance (adjacent subunits) (nm)	Distance restraint force constant (kJ/mol/nm2)
4QE9-0.8	2.58	1.59	500
4QE9-0.4	2.98	1.99	500
3LDC	3.66	2.6	500
3LDC+0.2	3.86	2.8	500
3LDC+0.3	3.96	2.9	500
3LDC+0.4	4.06	3.0	500
3LDC+0.5	4.16	3.1	500
1LNQ-0.2	3.8	2.6	500
1LNQ	4.0	2.8	500
1LNQ+0.2	4.2	3.0	500
1LNQ+0.4	4.4	3.2	500
1LNQ+0.6	4.6	3.4	500

Supplementary Table 7. List of simulated systems (water in the SF).

System name	Restrained P19 CA-CA distance (opposite subunits) (nm)	Restrained P19 CA-CA distance (adjacent) (nm)	Restrained F97 CA-CA distance (opposite subunits) (nm)	Restrained F97 CA-CA distance (adjacent subunits) (nm)	Distance restraint force constant (kJ/mol/nm2)
1LNQ+0.2	4.5	3.2	4.2	3.0	1000
1LNQ+0.4	4.7	3.4	4.4	3.2	1000
1LNQ+0.6	4.9	3.6	4.6	3.4	1000

Supplementary Table 8. List of simulated systems (voltage 150 mV).

System name	Restrained P19 CA-CA distance (opposite subunits) (nm)	Restrained P19 CA-CA distance (adjacent) (nm)	Restrained F97 CA-CA distance (opposite subunits) (nm)	Restrained F97 CA-CA distance (adjacent subunits) (nm)	Distance restraint force constant (kJ/mol/nm2)
4QE9-0.6	2.87	1.85	2.78	1.79	1000
4QE9-0.4	3.07	2.05	2.98	1.99	1000
4QE9	3.47	2.45	3.38	2.39	1000
3LDC	3.6	2.55	3.66	2.6	1000
3LDC+0.2	3.8	2.75	3.86	2.8	1000
3LDC+0.3	3.9	2.85	3.96	2.9	1000
1LNQ	4.3	3.0	4.0	2.8	1000
1LNQ+0.2	4.5	3.2	4.2	3.0	1000
1LNQ+0.4	4.7	3.4	4.4	3.2	1000
1LNQ+0.6	4.9	3.6	4.6	3.4	1000

Supplementary Table 9. List of simulated systems (NaK2K channel).

System name	Restrained A92 CA-CA distance (opposite subunits) (nm)	Restrained A92 CA-CA distance (adjacent subunits) (nm)	Distance restraint force constant (kJ/mol/nm2)
3OUF-0.3	1.37	0.88	1000
3OUF-0.2	1.47	0.98	1000
3OUF-0.1	1.57	1.08	1000
3OUF	1.67	1.18	1000
3OUF+0.1	1.77	1.28	1000
3OUF+0.2	1.87	1.38	1000
3OUF+0.3	1.97	1.48	1000
3OUF+0.4	2.07	1.58	1000
3OUF+0.5	2.17	1.68	1000

Supplementary Table 10. List of simulated systems (TRAAK channel).

System name	Restrained A270 CA-CA distance (nm)	Restrained T277 CA-CA distance (nm)	Restrained A162 CA-CA distance (nm)	Distance restraint force constant (kJ/mol/nm2)
4I9W-0.2	2.0	1.2	1.7	1000
4I9W	2.2	1.4	1.9	1000
4I9W+0.3	2.5	1.7	2.2	1000
4I9W+0.5	2.7	1.9	2.4	1000
4I9W+0.7	2.9	2.1	2.6	1000
4I9W+0.9	3.1	2.3	2.8	1000
4I9W+1.1	3.3	2.5	3.0	1000
4I9W+1.3	3.5	2.7	3.2	1000

SUPPLEMENTARY REFERENCES

1. Y. Jiang, *et al.*, Crystal structure and mechanism of a calcium-gated potassium channel. *Nature* **417**, 515–522 (2002).

2. X. Tao, R. K. Hite, R. MacKinnon, Cryo-EM structure of the open high-conductance Ca2+-activated K+ channel. *Nature* **541**, 46–51 (2017).

3. R. K. Hite, *et al.*, Cryo-electron microscopy structure of the Slo2.2 Na+-activated K+ channel. *Nature* **527**, 198–203 (2015).
