# Supplementary material for: Molecular mechanism of a potassium channel gating through activation gate-selectivity filter coupling
Source: Nat Commun. 2019 Nov 26;10:5366. doi: 10.1038/s41467-019-13227-w (PMC6879586; doi:10.1038/s41467-019-13227-w)
Supplement: Supplementary file 8 — Description of Additional Supplementary Files [file 41467_2019_13227_MOESM8_ESM.pdf]

Title: Supplementary Movie 1.
Description: Conformations of MthK with varying degrees of the AG opening, obtained with the AMBER force field.

Title: Supplementary Movie 2.
Description: Conformations of MthK with varying degrees of the AG opening, obtained with the CHARMM force field.

Title: Supplementary Movie 3.
Description: Collective motion of MthK that maximally correlates with T59 widening, obtained with the functional mode analysis in the CHARMM force field.

Title: Supplementary Movie 4.
Description: Collective motion of MthK that maximally correlates with T59 widening, obtained with the functional mode analysis in the AMBER force field.

Title: Supplementary Software.
Description: FORTRAN code analysing various properties of GROMACS (.xtc) trajectories of potassium channels - number of ion permeation events, occupancy of each site in the selectivity filter, and flips of carbonyl oxygens.
